# Etymologia: *Anopheles*

**DOI:** 10.3201/eid1809.ET1809

**Published:** 2012-09

**Keywords:** etymologia, Anopheles, malaria, mosquitoes, Meigen

## *Anopheles* [ə-nofʹə-lēz]

From the Greek *an* (“not”) + *ophelos* (“benefit”), a genus of mosquitoes, many species of which are vectors of malaria. *Anopheles* was first described by German entomologist Johann Wilhelm Meigen in 1818. Although some sources translate *Anopheles* as “harmful,” it would be decades before Ronald Ross showed in 1897 that these mosquitoes transmit malaria parasites, and Meigen was most likely using *Anopheles* in a more literal interpretation as “useless.” That said, the connotation of “harmful” was prophetic in describing a mosquito that, even today, is indirectly responsible for ≈1 million deaths per year.
